# Oral invasive procedures in Glanzmann thrombasthenia: a retrospective observational study

**DOI:** 10.1016/j.rpth.2024.102619

**Published:** 2024-11-04

**Authors:** Maxime Delarue, François Severac, Martine Soell, Léa Pierre, Dominique Desprez, Fabien Bornert

**Affiliations:** 1 University of Strasbourg, Faculty of Dental Surgery, Strasbourg, France; 2 University Hospital Strasbourg, Department of Oral Surgery, Strasbourg, France; 3 University of Strasbourg, Faculty of Medicine, Strasbourg, France; 4 University Hospital Strasbourg, Department of Public Health, GMRC, Strasbourg, France; 5 University Hospital Strasbourg, Dental Unit, Hôpital de Hautepierre, Strasbourg, France; 6 University Hospital Strasbourg, Department of Haemostasis, Regional Inherited Bleeding Diseases Center, Hôpital de Hautepierre, Strasbourg, France; 7 INSERM (French National Institute of Health and Medical Research) UMR 1260, Regenerative Nanomedicine, CRBS, Strasbourg, France

**Keywords:** blood platelet disorders, dental scaling, hemorrhage, oral, rFVIIa, surgery, thrombasthenia, tooth extraction

## Abstract

**Background:**

Glanzmann thrombasthenia (GT) is a very rare autosomal inherited bleeding disease affecting megakaryocyte lineage with impacts on oral health such as gingival bleeding, which requires specific management protocols. Very few clinical cases have been published in the dental and hematologic literature.

**Objectives:**

This study focuses on a series of 21 patients affected specifically by GT and their hemorrhagic prophylaxis management with the use of recombinant activated factor VII (rFVIIa) for dental extractions and full-mouth debridement.

**Methods:**

Data were collected from medical and dental records. rFVIIa was administered prophylactically for oral procedures, following a standardized protocol. Each sessions were performed by experienced oral surgeons, and outcomes were analyzed with a focus on bleeding complications and adverse events.

**Results:**

Forty-one full-mouth debridements and 176 dental extractions were performed during 102 sessions of dental care in an outpatient setting. A total of 226 injections of rFVIIa was delivered. The mean number of injections was 2.2 (range, 1-4) per dental procedure. The overall rate of bleeding complications was 5.9% (n = 6). All 6 hemorrhagic complications were classified as minor bleeding. No thromboembolic event or allergic reaction was observed.

**Conclusion:**

The data presented in this retrospective observational study support the efficacy and safety of rFVIIa for the prevention of bleeding during invasive dental procedures in patients affected by GT. The rFVIIa protocol presented here seems to be a safe and efficient protocol for the prevention of bleeding during invasive oral procedures.

## Essentials

• Glanzmann thrombasthenia (GT) is a rare platelet function disorder resulting in severe bleeding.
• Recombinant activated factor II (rFVIIa) was used for oral bleeding prophylaxis during invasive dental procedures in patients with GT.
• In 102 dental care sessions, there were 6 minor hemorrhagic complications (94% success rate).
• rFVIIa is efficacious and safe for the prevention of bleeding during invasive dental procedures.

## 1 Introduction

Glanzmann thrombasthenia (GT) is an autosomal inherited bleeding disorder affecting megakaryocyte lineage. Platelet aggregation disorder is caused by a quantitative deficiency and/or qualitative defects in transmembrane protein integrin αIIbβ3, also known as GPIIb/IIIa. This integrin constitutes a glycoprotein complex, and its activated form serves as the predominant receptor on the platelet membrane for binding fibrinogen. The αIIb and β3 subunits of the receptor are encoded by different genes located on the same chromosome (chromosome 17). Homozygous or heterozygous mutations of either the αIIb or β3 gene are responsible for quantitative or qualitative deficiencies seen in GT. Several genotypes and phenotypes are associated with GT. Three types of GT have been described: type I is a severe GPIIb/IIIa deficiency (expression levels <5%); type II is a moderate GPIIb/IIIa deficiency (5%-20% expression); and patients with higher expression (>20%) but with a dysfunctional GPIIb/IIIa are classified as having the variant form of GT [[1], [2], [3], [4]].

The Swiss pediatrician Eduard Glanzmann was the first to discover this primary hemostasis disorder in 1918. GT is considered a rare disease and is estimated to occur in 1 in 1,000,000 in the general population. Despite worldwide distribution, a large proportion of the cases have been described in selected ethnic groups in which there is a high rate of consanguinity: French Manouche Gipsy, South Indian Hindus, Iraqi Jews, and Jordanian nomadic tribes [4]. In France, with a population of approximately 67 million people, the prevalence is estimated to be about 300 patients [5].

**Figure 1:**
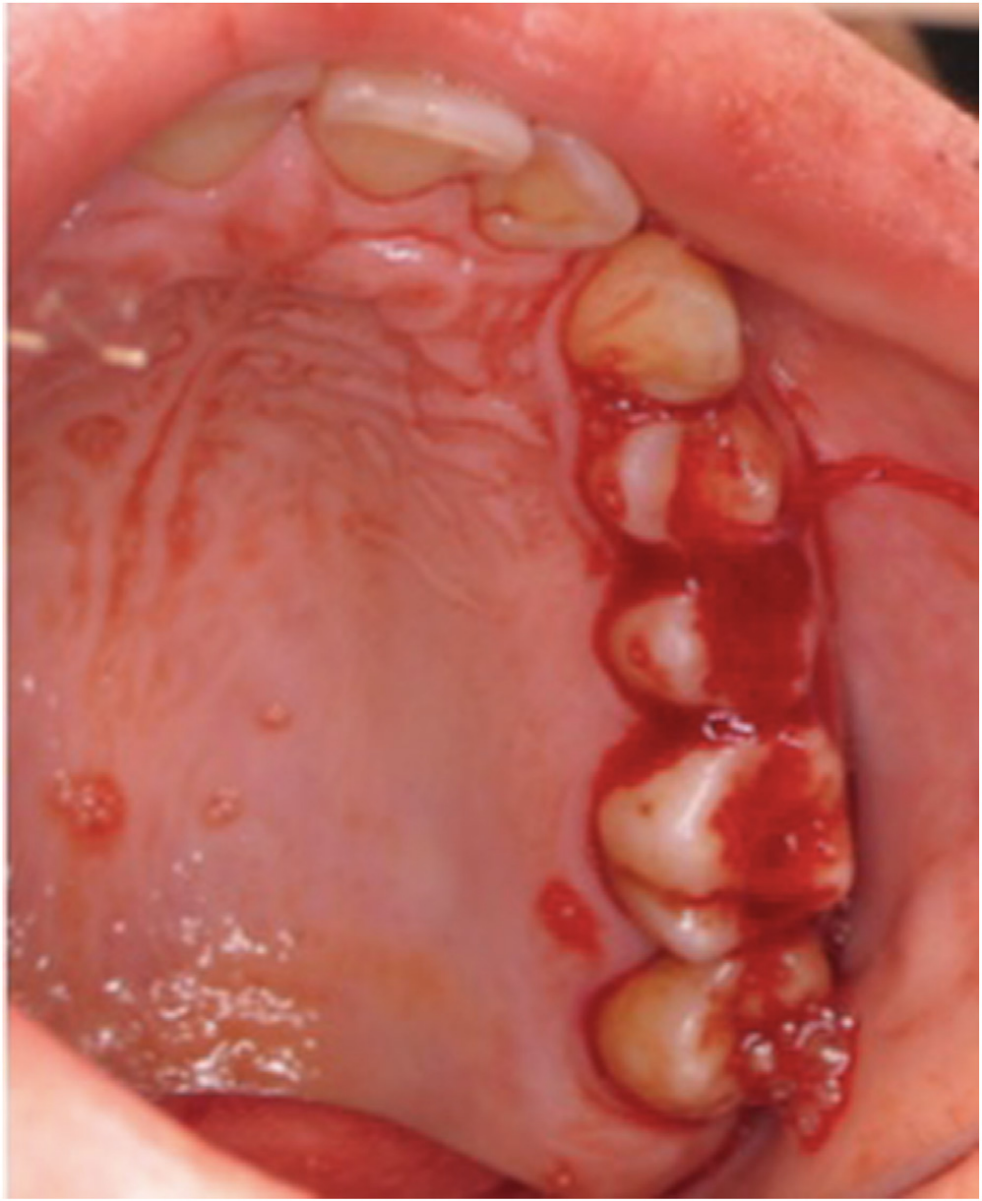
Spontaneous gingival bleeding in a 30-year-old woman affected by Glanzmann thrombasthenia type 1.

GT is characterized by spontaneous mucocutaneous bleeding and an exaggerated response to trauma mostly starting during early childhood. Patients with GT have spontaneous, lifelong mucocutaneous bleeding as epistaxis, menorrhagia, gastrointestinal bleeding, and gingival bleeding (Figure 1) [6]. The patients usually have chronic gingivitis and periodontal disease associated with chronic blood loss. Patients with GT are usually are hesitant to brush their teeth and are generally poorly compliant in dental maintenance, despite therapeutic education focused on dental hygiene [7,8]. Hemorrhagic complications after oral procedures are frequent. The oral cavity has an important network of blood vessels supporting many physiological activities (such as chewing, swallowing, and speaking). The achievement of good surgical hemostasis in the mouth may sometimes be difficult because of the presence of inflammatory tissues and the limited space of the operating field, making it harder to perform manual hemostatic compression with gauze and antifibrinolytic agents [9].

Platelet transfusion has been considered the standard treatment for preventing or controlling severe hemorrhage in GT. However, platelet transfusion can have detrimental effects, including the production of GPIIb/IIIa isoantibodies or anti-human leukocyte antigen (HLA) antibodies and/or platelet refractoriness. Recombinant activated FVII (rFVIIa) has been proposed as an alternative treatment to platelet transfusion [10].

In 1996, a patient with GT experiencing severe epistaxis was the first to be successfully treated with rFVIIa (NovoSeven, Novo Nordisk) [11]. rFVIIa is a prohemostatic agent approved by the European Medicines Agency in 2004 for the treatment of bleeding episodes and for the prevention of bleeding during surgery or invasive procedures in patients with GT with alloimmunization to platelet antigens, or with past or present refractoriness to platelet transfusions, or where platelets are not readily available. Since then, rFVIIa has been widely used in patients with GT either for bleeding events or as prophylaxis for all kinds of surgeries [12].

No guidelines exist regarding the use of rFVIIa in dental practice. This study is the first to specifically examine a large series of patients with GT with a protocol using rFVIIa in the prophylaxis of bleeding after tooth extraction or full-mouth debridement (FMD). We report here our experience with patients with GT concerning oral surgery procedures. The aim of this study was the evaluation of the efficiency and security of a hemostasis protocol using rFVIIa for outpatient invasive oro-dental care (dental extraction and FMD) in patients affected by GT.

## 2 Methods

### 2.1 Study design

This retrospective observational study was performed in the University Hospital of Strasbourg through a close collaboration between the Oral Surgery Department and the Resources and Competence Center for Constitutional Hemorrhagic Diseases (RCCHD) from January 1, 2008 to December 31, 2021. Data were collected by cross-referencing medical and dental records databases. Patient consents were previously collected. The eligibility criteria were patients affected by GT defined by a complete or partial qualitative or quantitative reduction of GPIIb/IIIa sites and genotyping of the receptor. Patients with acquired thrombasthenic states caused by medications or hematologic and autoimmune disorders were included. Patients with GT were treated using an rFVIIa prophylaxis protocol for tooth extraction and/or FMD. The RCCHD of Strasbourg database included 41 patients with GT. Demographic data including patients’ age (years), sex, weight (kilograms), GT type, history of spontaneous gingivorrhagia, and antiplatelet antibodies status (anti-HLA antibodies, anti-GPIIb/IIIa antibodies, or both) were also identified. Dental data included number of sessions, number of teeth extracted per session, type of teeth, and presence and type of complications (infectious or hemorragic). Hemorragic episodes data were classified into 3 categories:

1. No hemorragic episode;
2. Minor bleeding defined by new local hemostasis maneuver and/or rFVIIa injection in an outpatient manner;
3. Major bleeding defined by reintervention with local hemostasis and/or rFVIIa injection with hospitalization and/or platelet transfusion and/or red blood cell transfusion.

Concerning the rFVIIa protocol, different medical data included number of rFVIIa injections per session, dosage unit of the rFVIIa injection, and thromboembolic and allergic events. Data were anonymized and collected using Microsoft Excel (Microsoft Corp).

In this study, effectiveness was defined as the absence of hemorrhagic complications or adverse events in the management of tooth extractions and FMD in patients affected by GT.

### 2.2 Protocol

Tooth extraction and FMD were scheduled in an outpatient setting at the beginning of the week (Monday or Tuesday morning). Invasive dental procedures were performed by an oral surgeon experienced in managing patients affected by bleeding disorders.

Preoperative medications were:

• Antibiotics: oral amoxicillin 1 g starting 1 hour before surgery and 1 g 3 times a day for 7 days. In case of allergy, oral clindamycin 600 mg was prescribed 1 h before surgery and 600 mg 2 times a day for 7 days. The doses were adjusted for children relative to their weight.
• Per oral acetaminophen 1 g, once every 6 hours, for a maximum of 7 days. The doses were adjusted for children relative to their weight.
• Mouthwash (chlorhexidine 0.12%) 48 hours before surgery and 48 hours after surgery for 7 days.
• Per oral tranexamic acid (0.1 g/mL), an antifibrinolytic agent, 10 minutes before the surgical procedure and 12 and 24 hours after surgery.

rFVIIa (NovoSeven) was administered by an intravenous bolus in a single dose, delivered 10 minutes before surgical procedure (T1) in the Oral Surgery Unit by a specialized nurse under the supervision of a physician experienced in the treatment of bleeding disorders. Doses were calculated from the weight of the patients, with a dose range 80 to 120 μg/kg bodyweight according to the European Medicines Agency notice [13].

Local hemostasis procedures included local anesthesia (articaine 4% w/v with 1:200,000 epinephrine), supragingival scaling with limited gingival trauma, placement of intra-alveolar hemostatic resorbable agent (Pangen, Urgo Medical) or Surgicel (Ethicon), and resorbable stitches. Local compression with gauze and tranexamic acid was performed for at least 10 minutes after the surgical procedure.

An FMD is a nonsurgical procedure that helps to remove extensive plaque and tartar build-up from the teeth and under the gums. It consists ultrasonic and manual maxillo-mandibular debridement performed to remove supra- and infra-gingival tartar.

Monitoring for at least 30 minutes was required before the patient returned to hematology unit. After control of hemostasis, a second dose of rFVIIa was injected intravenously 2 hours after the surgical procedure (T2).

**Figure 2:**
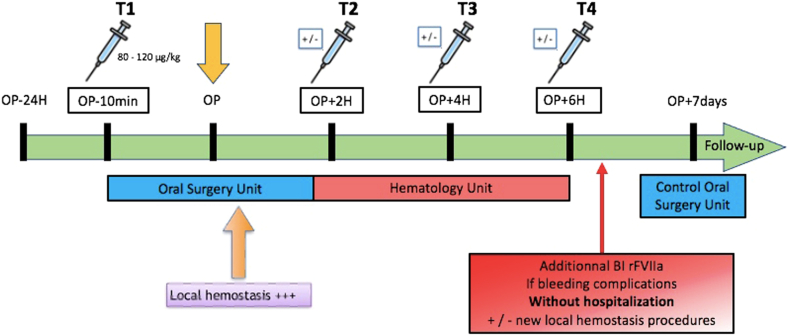
Management of hemostasis protocol with recombinant activated factor II injection for tooth extraction and full-mouth debridement. BI, bolus injection; OP, operation; T, time.

If bleeding persisted, a third dose was injected 4 hours after surgical extraction (T3). If hemostasis control was not satisfactory, a fourth injection was transfused 6 hours after surgical extraction (T4). After local hemostasis control was performed, the patient could return home. A 7-day follow-up appointment was scheduled to check for good healing of the wound (Figure 2).

### 2.3 Ethics approval and informed consent

All procedures were followed in accordance with the ethical standards of the responsible committee on human experimentation (institutional and national) and with the Helsinki Declaration of 1975, as revised in 2008. Informed consent was obtained from all patients or from parents or other legal guardians for inclusion in the study. Ethics approval was obtained from ethic committee of Faculty of Medicine, University of Strasbourg, France (Reference: CE-2022-60).

### 2.4 Data analysis

The demographic and clinical characteristics of patients are described using counts and percentages for categorical variables and median with IQR for continuous variables. Because it was more informative, the number of doses per procedure was presented using mean and range. Given that patients benefited from several procedures (102 procedures for 21 patients), comparisons at the procedural level were performed using mixed models with a random patient effect to account for the repetition of data for the same subject. Comparisons of counting variables (number of injections or number of extractions) were performed using a quasi-Poisson mixed model and comparison of postoperative bleeding with a logistic regression mixed model. Correlation between the number of rFVIIa injections and the number of dental extractions was assessed using the repeated-measures correlation coefficient [14]. Statistical analyses were performed using R version 4.1.1 (R Core Team, 2021). A *P* value < .05 was considered statistically significant.

## 3 Results

### 3.1 The GT Registry

**Figure 3:**
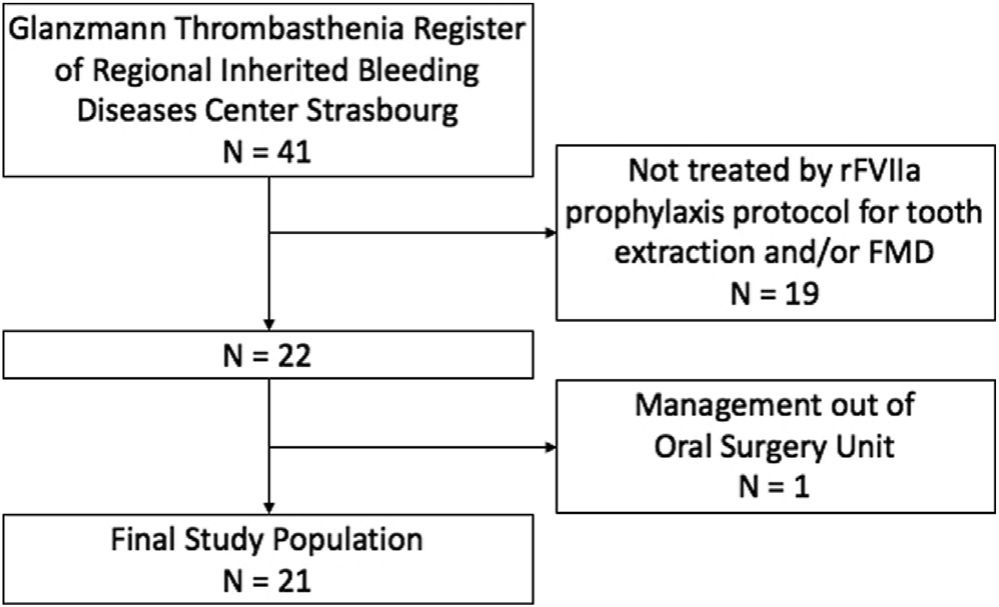
Study population flowchart. FMD, full-mouth debridement, rFVIIa, recombinant activated factor II.

This retrospective study included 21 patients with GT (Figure 3): 10 women and 11 men aged between 8 and 70 years at first admission (median age: 28; IQR: 21-40), who underwent dental extraction and/or FMD at the Oral Surgery Unit-University Hospital of Strasbourg, between January 1, 2008 and December 31, 2021. Nineteen patients were diagnosed with type I GT and 2 with type III. Fifteen patients (71%) had antiplatelet antibodies, 12 (57.1%) of which were against GPIIb/IIIa and 5 (23.8%) against HLA (with 2 patients having antibodies against both). Their weight ranged from 29 kg to 124 kg at first admission (median weight: 81; IQR: 67-105). Eighteen patients (85.7%) reported having previous gingival bleeding episodes that sometimes required emergency management. Forty-one FMDs and 176 dental extractions were performed during 102 sessions of dental care (Table 1). One hundred eight maxillary teeth and 68 mandibular teeth were extracted. The distribution of dental extractions by type of tooth is described in Table 1.

**Table 1:** Description of types of teeth extracted during the study.

Types of teeth	Maxillary	Mandibular	Total
Incisor	19	9	28
Canine	12	5	17
Premolar	30	20	50
Molar	45	33	78
Deciduous tooth	2	1	3
Impacted tooth	0	0	0
Total	108	68	176

### 3.2 Efficacity and safety of rFVIIa transfusion

Eight complications requiring postoperative management were observed during the 102 sessions: 2 infections and 6 postoperative bleeding episodes. The overall efficacy of rFVIIa was 94.1%. The global rate of bleeding complications was 5.9%. All 6 hemorrhagic complications were classified as minor bleeding (Table 2). Four patients were managed with local hemostasis with gauze compression combined with local application of tranexamic acid. Two patients (at 2 different sessions) required 2 more injections each the day after the dental procedure. No patient was hospitalized or required a platelet transfusion or red blood cell transfusion. No thromboembolic events or allergic reactions were observed.

**Table 2:** Description of the 6 cases of postoperative bleeding episodes during the study.

Patient	Age (y)	Dental procedure	Total injection protocol (before bleeding at home)	Postoperative management
1	32	Extraction 5 teeth: 2M, 2PM, 1C	2	Packing^a^ 15 d after dental procedure
2	47	FMD only	4	Packing^a^ the following day very important gingival inflammation is noted
	55	Extraction 3 teeth: 2M, 1C	3	2 supplementary rFVIIa injections the following day; return home
3	26	Extraction 3 teeth: 3M	4	Packing^a^ 2 d after dental procedure
4	15	FMD only	2	Packing^a^ the following day
5	46	FMD associated Extraction 2 teeth: 2PM	2	2 supplementary rFVIIa injection the following day; return home

C, canine; FMD, full-mouth debridement; M, molar; PM, premolar; rFVIIa, recombinant activated factor II.

a Packing: local hemostasis with gaze compression associated to tranexamic acid.

### 3.3 rFVIIa dosing

**Figure 4:**
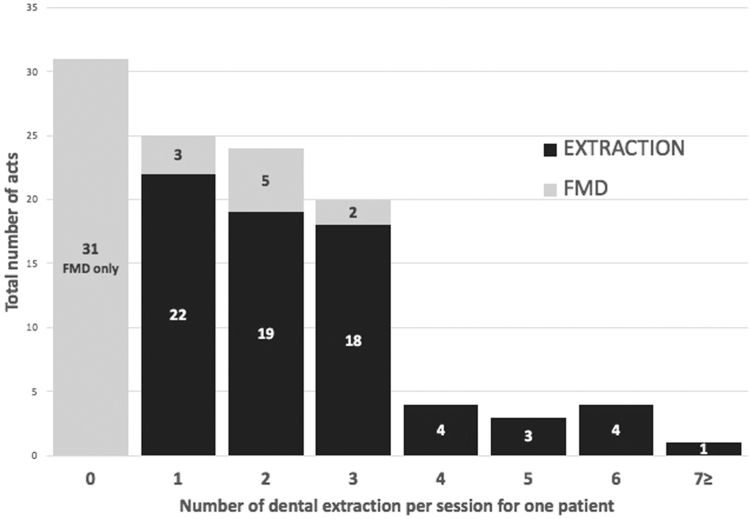
Distribution of dental procedures in Glanzmann thrombasthenia patients along 102 sessions. FMD, full-mouth debridement.

A total of 226 injections was delivered during the 102 sessions. The mean number of injections was 2.2 (range: 1-4) per dental procedure. All patients for all sessions (100%, n = 102) received the first intravenous rFVIIa transfusion (T1) 10 minutes before dental extraction or FMD, corresponding to a mean T1 dose of 118 μg/kg. Eighty-three percent (83.3%, n = 85) of patients with GT received a second rFVIIa transfusion (T2) 2 hours after the dental procedure, corresponding to a mean T2 dose of 112 μg/kg. Thirty-three percent (33.3%, n = 34) of patients received a third rFVIIa transfusion (T3) 4 hours after the dental procedure, corresponding to a mean T3 dose of 118 μg/kg. In only 5 sessions (4.9%, n = 5) patients needed a fourth rFVIIa transfusion (T4) 6 hours after the oral procedure because of persistent bleeding, corresponding to a mean T4 dose of 160 μg/kg (Figure 4). The mean total dose was 116 μg/kg per injection. The median dose per infusion and the median cumulative rFVIIa dose varied depending on the type of procedure performed (Table 3).

**Table 3:** Description of the doses and efficacy of rFVIIa according to the type of invasive oral procedure.

Oral surgery type	rFVIIa dosing				rFVIIa efficacy		
	No. of procedures	Dose per infusion (μg/kg), median (IQR)	Total dose per procedure (μg/kg), median (IQR)	No. of doses per procedure, mean (range)	No. of procedures	Minor bleeding	Efficacy (%)
All dental procedures	102	111 (95-172)	215 (165-406)	2.2 (1.0-4.0)	102	6	94.1
All dental extractions (with FMD sometimes associated)	71	120 (97-182)	270 (186-421)	2.4 (1.0-4.0)	71	4	94.4
FMD alone	31	100 (87-118)	157 (97-200)	1.8 (1.0-4.0)	31	2	93.5
Dental extraction alone (without FMD associated)	61	133 (99-182)	240 (185-500)	2.4 (1.0-4.0)	61	3	95.1
Dental extraction (associated with FMD)	10	111 (95-126)	271 (199-333)	2.5 (2.0-3.0)	10	1	90.0
Extraction >3 teeth	12	159 (140-182)	543 (264-545)	2.7 (1.0-4.0)	12	1	91.7
Extraction ≤ 3 teeth (without FMD associated)	49	111 (96-172)	220 (183-402)	2.3 (1.0-4.0)	49	2	95.9
Extraction ≤ 3 teeth (associated with FMD)	59	111 (96-170)	222 (185-402)	2.3 (1.0-4.0)	59	3	94.9

FMD, full-mouth debridement; rFVIIa, recombinant activated factor II.

### 3.4 Risk factors

We observed a moderate positive correlation between the number of rFVIIa injections and the number of dental extractions (Rho = 0.36 [0.10; 0.58], *P* = .009). The number of doses administered for 1 to 3 dental extractions was statistically lower than for >3 extractions (mean 2.1 ± 0.5 vs 2.7 ± 0.6, respectively, *P* < .001). However, there was no association between the number of extractions and the occurrence of postoperative bleeding (mean 2.4 ± 1.5 vs 3.2 ± 1.3, *P* = .191). There was also no association between the number of rFVIIa injections and the occurrence of postoperative bleeding (mean 2.4 ± 0.6 vs 2.7 ± 1.0, *P* = .239).

The mean number of rFVIIa perfusions during FMD was statistically lower than for a dental extraction procedure (mean 1.8 ± 0.9 vs 2.4 ± 0.6, *P* < .001). Nevertheless, no association was found between the type of dental procedure (FMD or dental extraction) and the occurrence of postoperative bleeding (6.5% vs 5.6%, *P* = .871).

## 4 Discussion

This study is the first dedicated to invasive dental procedures in a large series of GT patients with a protocol using rFVIIa for the prophylaxis of oral bleeding. The majority of previous reports are clinical cases [[15], [16], [17], [18]] or case series [6,12,19] for the dental management of patients with GT because GT is a low prevalent disease. Use of rFVIIa for local hemostasis as a bleeding prophylaxis had a high success rate superior to 94%, with only 6 minor hemorrhagic complications in 102 dental care sessions. No hospitalizations were necessary because the minor complications could be managed at home. Four patients needed a hemostasis procedure with the use of tranexamic acid used locally with compression for 10 minutes. Only 2 patients needed a complementary rFVIIa transfusion with local hemostasis, but they were able to go home the same day. In addition, no biological monitoring was necessary. This protocol requires a close collaboration between oral surgery and hematology teams. It is difficult to compare our results with those of other studies because most studies have evaluated the effectiveness of rFVIIa on the bleeding prophylaxis of different types of surgical procedures [20]. The originality of this study is its focus only on invasive dental surgery procedures, which are considered minor surgical procedures. In different studies, minor surgery is defined as any invasive operative procedure in which only skin, mucous, membranes, or superficial connective tissue are manipulated [10,20,21]. For dental extraction, the situation is slightly different because alveolar bone is directly exposed during the surgery. However, we noticed a quite similar success rate for dental surgical procedures than Poon et al., who showed an 88% success rate for a minor surgical procedure. The effectiveness was generally similar irrespective to refractoriness/antibody status of the patients [22].

Recht et al. [20] reported 100% effectiveness of rFVIIa during 134 minor surgical procedures, including a majority of dental procedures, without specifying the type of procedure or surgical protocol. The median dose of rFVIIa administered by infusion in minor surgical procedures treated with rFVIIa was 100 (90-140) μg/kg. The number of doses per admission with rFVIIa was 2.5 (2.0-3.0), relatively similar to our study. However, it is important to note that variable definitions and scales for evaluating efficacy as well as variable time periods after bleeding used by investigators may also limit comparisons [20]. In our retrospective study, the criteria for evaluating the efficacy of rFVIIa share similarities with the scale used by Poon et al. [20,22] in different studies [21], but differences were noted. The scale implemented in this study stands out for its specificity to oral invasive procedures and the inclusion of additional details regarding interventions necessary to evaluate the postoperative oral bleeding severity and the absence of adverse effects induced by rFVIIa.

Based on these results, rFVIIa at 120 μg/kg at intervals of 2 hours should be used, at least at the beginning of treatment. The subsequent number of doses required should be determined by the clinical situation, and dosing should be continued until hemostasis is achieved [23]. Normally, 2 or 3 doses are required. It is necessary to maximize the rFVIIa dose to 120 μg/kg to achieve a minimum of postoperative bleeding at home. In case of clinical ineffectiveness, the dosage of rFVIIa should be adapted to clinical response by increasing the dose in stages. A high dose of rFVIIa induces the thrombin burst converting fibrinogen to fibrin at the surgical sites in patients with GT because they have normal plasma clotting factors. Thrombin is not only responsible for the formation of fibrin, but also for the stabilization of fibrin by activating factor XIII and the inhibition of fibrin degradation by activating thrombin-activatable fibrinolysis inhibitor [19].

The association of general hemostatic agents with local hemostasis procedures constitutes a secure protocol to prevent oral bleeding complications for invasive oral procedures with a low rate of side effects [24,25]. Tranexamic acid is an antifibrinolytic agent widely used as an adjunctive treatment in all constitutional and acquired hemostasis deficiencies to prevent or attenuate bleeding maintained by fibrinolytic enzymatic activity. Its effectiveness in reducing perioperative bleeding in different types of surgeries has been widely demonstrated [18,19,23,24]. Commonly in oral surgery, its use is local either as a mouthwash or by soaking compresses to compress the hemorrhagic oral site [[26], [27], [28]]. Its use orally or parenterally must therefore be prescribed with caution, particularly in patients at high thrombotic risk and especially in concert with rFVIIa [18,19,29,30]. The overall prophylactic tendency is likely to decrease the amount and duration of coagulation factor substitution and to focus the local management of surgical wounds with local haemostatic biomaterials and specific techniques of compression [29].

Use of rFVIIa offers several benefits over platelet transfusion. There is no need for difficult assessments of antibody or refractoriness status, which can be time-consuming in emergency situations [28]. rFVIIa prevents the risk of alloimmunization and refractoriness associated with platelet transfusions. There is a substantial risk of transfer of alloantibodies to the fetus via the placenta following platelet transfusions in GT pregnant women, resulting in potentially fatal neonatal bleeding [4,30]. In addition, platelets may not be immediately or readily available in all clinics and are associated with the risk of blood-born pathogen transmission [31]. For these reasons, European guidelines suggest limiting the use of prophylactic platelet transfusion and using therapeutic platelet transfusion for cases unresponsive to other hemostatic measures [26,32]. Avoiding antiplatelet immunization by replacing platelet concentrates with rFVIIa is therefore of real interest. Moreover, rFVIIa has no immunologic side effect, but rare thromboembolic events have been described in some particular medical conditions with a 1% to 2% frequency [21,22,33,34]. Only a few cases have been reported in the literature but none in our series [[35], [36], [37], [38]].

Dental prophylaxis has to be thoughtfully developed for patients with GT to limit chronic blood loss and control the escalation of health care costs associated with multiple rFVIIa infusions. In our series, 85.7% of the patients with GT had a history of gingivorrhagia. It is frequently the first reason for consultation. Patients with GT may avoid brushing their teeth due to the risk of bleeding, plaque formation, and oral blood phobia, resulting in recurrent and severe bleeding that may require iron supplementation or blood transfusion. This vicious cycle worsens as patients continue to neglect oral hygiene, enhancing dental care needs. The hematologist can more easily observe and monitor oral health to prevent invasive procedures. FMD, which involves supra- and subgingival ultrasonic and mechanical tartar removal in a single session, is a procedure associated with a moderate risk of hemorrhage due to its invasive and extensive nature. This procedure can lead to damage of blood vessels and is particularly exacerbated in cases of chronic gum inflammation. The primary objective of this treatment is to eliminate tartar and plaque deposits, which are major sources of gingival inflammation and gingival bleeding [39]. Rasaratnam et al. [39] published a Dental Bleeding Risk Assessment and Treatment Tool that stratifies patients with inherited bleeding disorders according to the perceived invasiveness of the dental procedure (noninvasive, mild, moderate, and highly invasive) and the severity of the bleeding disorder (mild, moderate, and severe) into 4 bleeding risk groups: no risk, low risk, moderate risk, and high risk, thus correlating the invasiveness of the dental treatment with the bleeding risk and the required hemostatic protocol. Dental extractions are considered a highly invasive procedure with high bleeding risk regardless the severity of the bleeding disorder [39,40]. Preoperatively, the benefit-risk balance must be measured between the number of dental extractions per session, their location, the invasiveness of the surgical procedure, the dose and number of rFVIIa injections, the thrombotic risk, and the occurrence of postoperative hemorrhage.

Subgingival debridement is considered a minimally invasive procedure with low to moderate hemorrhagic risk depending on the severity of the bleeding disorder. Our study showed that subgingival debridement, a common and essential procedure for periodontal health, can be considered an invasive procedure in the context of GT. rFVIIa transfusion is essential in the prevention of hemorrhagic complications from FMD in patients with GT. The average number of doses and the average cumulative dose of rFVIIa is slightly reduced compared to dental extractions, but we encountered 2 minor bleeding complications that occurred following subgingival debridement, which were easily managed.

## 5 Conclusion

The data presented in this retrospective observational study support the efficacy and safety of rFVIIa for the prevention of bleeding during invasive dental procedures in patients affected by GT.

Preventive dental care plays a crucial role in managing these patients. It is essential to provide them with dental hygiene advice and hygienic-dietary recommendations. Additionally, implementing biannual dental check-up visits is strongly recommended to ensure regular monitoring of oral health status and prevent potential complications.
